# Complete mitochondrial genome of the decomposer *Ptecticus tenebrifer* Walker, 1849 (Insecta: Diptera: Stratiomyidae)

**DOI:** 10.1080/23802359.2023.2184659

**Published:** 2023-03-10

**Authors:** Gilsang Jeong, Areum Han, Hyejin Kang, Yeo Won Jeong, Seon Deok Jin

**Affiliations:** a Division of Restoration Research, Research Center for Endangered Species, National Institute of Ecology, Youngyang-gun, Gyeongsanbuk-do, Republic of Korea; b Ecological Observation Team on Climate Change, National Institute of Ecology, Seocheon-gun, Chungcheongnam-do, Republic of Korea; c Department of Zoological Management, National Institute of Ecology, Seocheon-gun, Chungcheongnam-do, Republic of Korea; d Planning and Budget Dept., National Institute of Ecology, Seocheon-gun, Chungcheongnam-do, Republic of Korea

**Keywords:** *Ptecticus tenebrifer*, mitochondrial genome, organic waste decomposition

## Abstract

*Ptecticus tenebrifer* (Walker, 1849), which is endemic in East Asia, plays a critical role in natural processing of organic waste. In this study, we found that the complete mitochondrial genome of *P. tenebrifer* is 15681 bp in length obtained from the MGIseq system (MGI) with 150 bp paired-end reads. As with typical animal mitochondrial genomes, it contains 13 protein coding genes, 22 tRNA genes, and 2 rRNA genes. The phylogenetic analysis was performed using the maximum likelihood method with a bootstrap value of 500 and the Tamura-Nei model and reveals the close relationship with *P. aurifer.*

## Introduction

**Figure 1.**
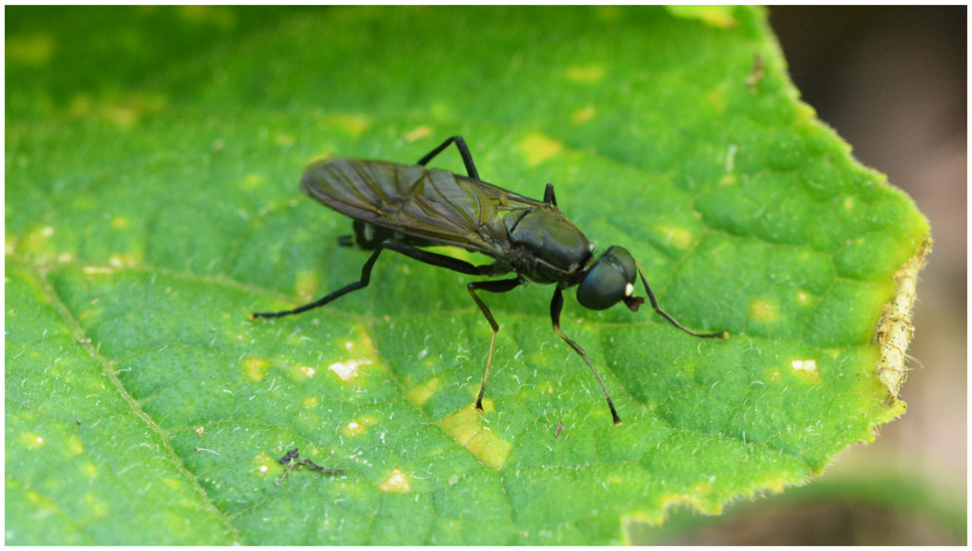
Photograph of *Ptecticus tenebrifer* (© Gilsang Jeong). Head dark brown to black; eyes bare. Antenna black but inner side of pedicel and flagellomeres 2–3 as well as basal third of arista more brownish; thorax dull dark brown to black. Wings brownish infumated. Legs chiefly black and black haired; Abdomen conspicuously clublike, constricted at base, black, but second segment translucent whitish with a differently shaped black dorsal mid spot (Rozkosny and Kovac 2000).

Nutrient cycling is an essential process for maintaining proper ecosystem functions (Jeong et al. 2021). The black soldier fly, *Hermetia illucens* (Linaeus, 1758) (Diptera: Stratiomyidae) has attracted worldwide attention for its role in digesting various types of organic waste, i.e. cycling nutrients (Kaya et al. 2021). However, other insects that play similar roles, such as *Ptecticus tenebrifer* Walker (Diptera: Stratiomyidae) (Figure 1), have been largely neglected. This insect species is endemic in East Asia, including South Korea. Its larvae can be easily found in various organic waste materials, over which the adults of the species are actively patrolling. The insect represents a valuable natural resource with multiple future applications. Furthermore, based on the Aichi target, genetic resources may be protected and benefits from such resources may be shared. In this regard, genetic information of endemic species becomes important.

## Materials and methods

Here, we report the first complete mitochondrial genome of *P. tenebrifer*, collected at Seocheon-gun(county), Chungcheongnam-do, Republic of Korea (36.03064 N, 126.72638 E) in August 2019. The specimen and gDNA were deposited at National Institute of Ecology (NIE hereafter) (https://www.nie.re.kr/contents/siteMain.do?mu_lang=ENG, Gilsang Jeong, gilsangj@nie.re.kr, voucher number: SC-5). Library preparation was performed using the MGIEasy DNA library prep kit (MGI). Sequencing was conducted on the MGIseq system (MGI) to generate 150-bp paired-end reads. To isolate the proper seed sequence for mitochondrial genome assembly using Novoplasty, trimmed reads were mapped to the Midori COI reference database (GB238, Uniq). Mapping coverage and depth at each locus were calculated using the genomecov module of Bedtools v2.27.1. A candidate seed longer than 150 bp was manually selected for mitochondrial genome assembly. Using candidate seeds, Novoplasty v4.2 was repeatedly applied with various K-value parameters until a complete circularized mitochondrial genome was obtained. The annotation module from Mitoz v2.3(or Mitos) was used to annotate and visualize the constructed mitochondrial genome.

For constructing the phylogenetic relationship among closely related species, we retrieved the 13 complete mitochondrial genome sequences from NCBI including *Apis mellifera* as an outgroup. They were aligned using MUSCLE followed by removing all gaps resulting in total of 10,596 bp on MEGA (ver.11) (Kumar et al. 2018). The maximum likelihood inference of 13 complete mitochondrial genome sequences was performed with a bootstrap value of 500 and the GTR + G (general time reversal + gamma) model on MEGA (ver.11) (Kumar et al. 2018). The resulting phylogenetic tree was visualized and modified on Treegraph 2 (2.15.0_887 beta) (Stöver & Müller 2010) and Inkscape (ver 1.2).

## Results

**Figure 2.**
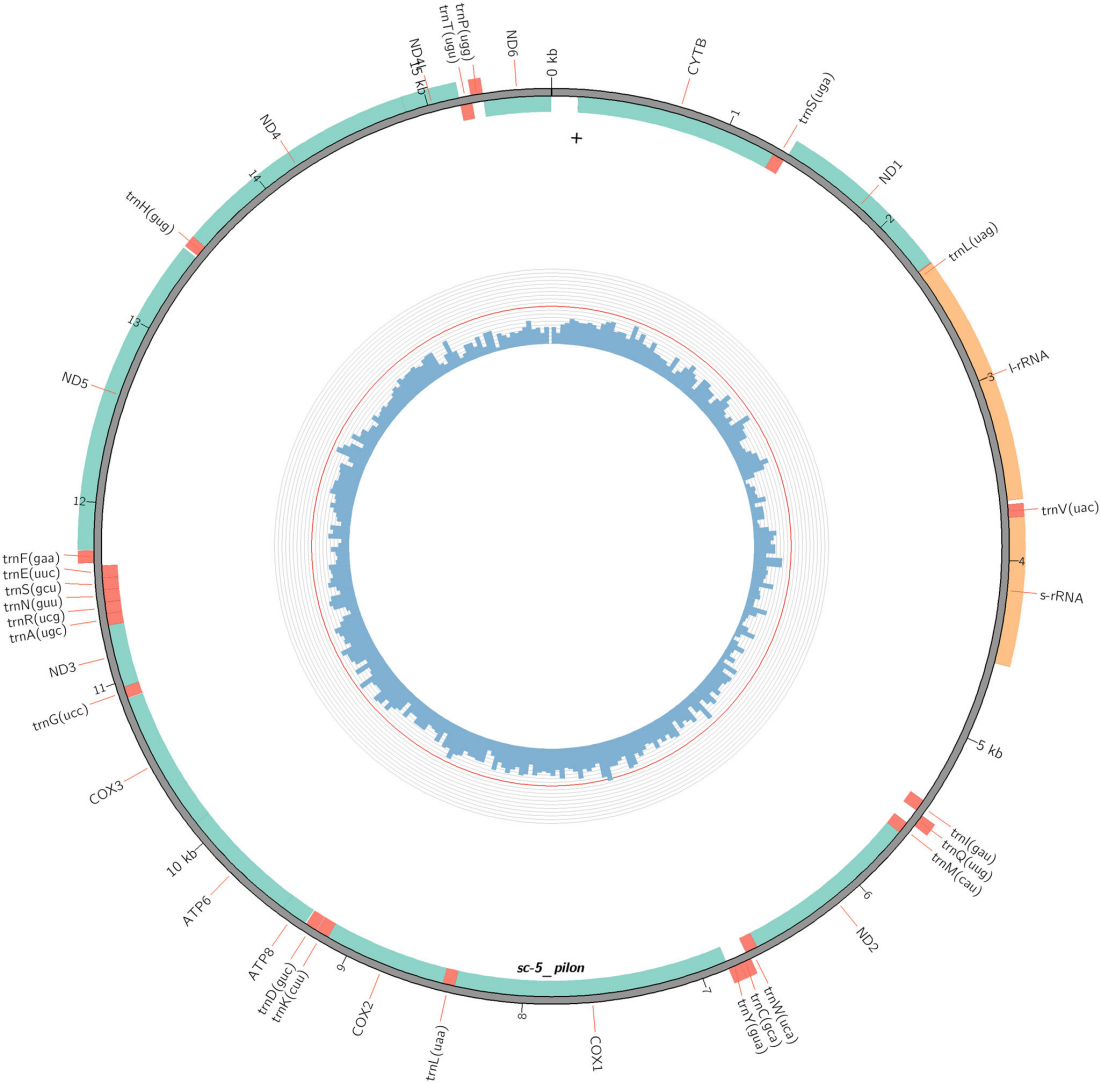
Physical circular map of the Mitochondrial genome of *Ptecticus tenebrifer.*

The complete genome of *P. tenebrifer* is 15,681 bp in length (GenBank accession number: MZ618618). Like other typical animal mitochondrial genomes, it contains 13 protein-coding genes, 22 tRNA genes, and 2 rRNA genes (Figure 2). The overall base composition is 38.01% A, 15.39% C, 9.88% G, and 36.71% T. The AT bias of the *P. tenebrifer* mitochondrial genome is significant, similar to those of *H. illucens* and *Nasimyia megacephala* (Yang and Yang 2010) (Qi et al. 2017; Hu and Yang 2021).

## Discussion and conclusion

**Figure 3.**
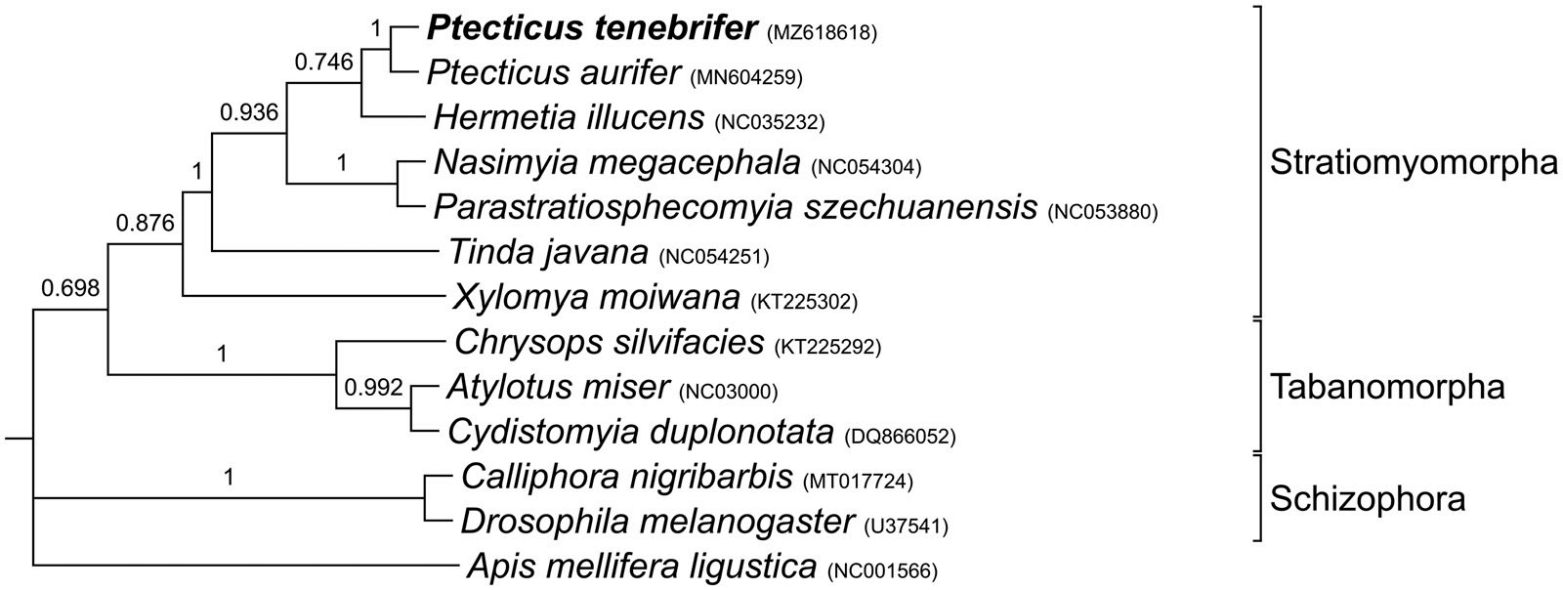
Phylogenetic relationship of *P. tenebrifer* among other Dipteran species. Further information on the mitogenomes used can be found in Cameron et al. 2007; Clary et al. 1982; Crozier & Crozier 1993; Hu and Yang 2021; Hu and Yang 2021; Hu and Yang 2021; Karagozlu et al. 2019; Stöver & Müller 2010; Qi et al. 2017; Wang et al. 2016; Zhan et al. 2020.

This phylogenetic analysis shows that Stratiomyomorpha and Tabanomorpha species are diverged from Schizophora counterpart at the basal node. Stratiomyomorpha and Tabanomorpha are monophyletic based on the full genome data (Figure 3). Most of Tabanomorpha species feed on nectar and pollen, though some Tabanidae species are known to feed on blood (Wiegmann et al. 2000). On the other hand, Statiomyomorpha species on the tree are not well described of their ecology except the *P. tenebrifer* and *H. illucens* having evolved the ability to decomposing organic wastes (Jeong et al. 2021). Schizophora species including *D. melanogaster* form the basal clade with A. mellifera (Figure 3). The phylogenetic resolution will be improved as more genome data are obtained. This study establishes the molecular taxonomic and phylogenetic placement of *P. tenebrifer* species. Except the organism of this study and *H. illucens*, life history of other Stratiomyomorpha species is largely unknown. Therefore, our study could be utilized for correct identification of the species as the future counter-measure of the current energy-consuming organic waste treatment, and for proving potential ecological roles of other closely related species.

## Data Availability

The genome sequence data that support the findings of this study are openly available from GenBank of NCBI at [https://www.ncbi.nlm.nih.gov] under the accession no. MZ618618. The associated BioProject, SRAs, and Bio-Sample numbers are PRJNA857657, SRR20077322, and SAMN29631756, respectively.
